# Biopsy-proven diagnoses associated with monodactylous longitudinal nail plate change in a diverse patient population: A retrospective cross-sectional study

**DOI:** 10.1016/j.jdin.2024.08.005

**Published:** 2024-09-01

**Authors:** Jeffrey N. Li, Ryan J. Scheinkman, Brian W. Morrison

**Affiliations:** Dr. Phillip Frost Department of Dermatology and Cutaneous Surgery, University of Miami Miller School of Medicine, Miami, Florida

**Keywords:** cutaneous oncology, diversity, erythronychia, longitudinal melanonychia, melanoma, nail cancer, pachyonychia, skin of color, squamous cell carcinoma, subungual

*To the Editor:* Monodactylous longitudinal nail plate change is a clinical phenomenon that implies an underlying pathology of the nail matrix. There are three well-described appearances, including longitudinal melanonychia (LM), longitudinal erythronychia (LE), and longitudinal pachyonychia (LPN). Each finding has a unique set of differential diagnoses. Prevalence data guiding decision-making in multiracial populations are understudied. Moreover, a recent analysis of Medicare utilization highlights the reluctance dermatologists have to perform nail unit biopsies.^1^ A lack of histological confirmation may further hinder the acquisition of accurate data. Here, we examine the prevalence of biopsy-proven diagnoses and the risk of malignancy in a multiracial population presenting with LM, LE, and LPN at our university hospital setting in Miami, Florida.

This was a retrospective single-center cross-sectional study of patients who underwent nail matrix biopsy for monodactylous LM, LE, or LPN between 01/01/2017 and 10/30/2023. All biopsies were reviewed by a board-certified dermatopathologist. Challenging cases were sent for consultation. Ethnicity and race were self-reported by patients.

We identified 128 biopsies from 127 patients. Of these patients, 71/128 had LM, 48/128 had LE, and 9/128 had LPN (Table I). LM diagnoses included melanocyte activation (35.21%), subungual lentigo (28.17%), nevus (16.9%), onychopapilloma (7.04%), and onychocytic matricoma (2.82%). Squamous cell carcinoma (SCC) in situ (2.82%) was the most common malignancy, and only 1 case of nail unit melanoma was identified. LE was associated with onychopapilloma (41.67%), verruca vulgaris (14.58%), digital mucous cyst (6.25%), chronic dermatitis (6.25%), glomus tumor (4.17%), and subungual fibrosis/scar (4.17%). Again, SCC was the most identified malignancy, with invasive SCC (6.25%) more common than SCC in situ (4.17%). The anatomical distribution for LE is demonstrated in Fig 1. For LPN, onychomatricoma accounted for 55.56% of cases, with 2 cases of onychocytic matricoma (22.22%) and 1 case of SCC (11.11%).

**Table I:** Patient demographics and diagnoses confirmed by nail matrix biopsies

	Number	Percentage/SD
Longitudinal melanonychia		
Age (mean, SD)	50.41	16.85
Gender (*n*, %)		
Male	28	39.44%
Female	43	60.56%
Ethnicity (*n*, %)		
White	10	14.08%
Hispanic or Latino	39	54.93%
Black or African American	15	21.13%
Asian	2	2.82%
More than one race	3	4.23%
Unknown or Not Reported	1	1.41%
Diagnosis (*n*, %)		
Melanocyte activation	25	35.21%
Nonmelanocytic tumors		
SCCIS	2	2.82%
Onychopapilloma	5	7.04%
Onychomatricoma	1	1.41%
Onychocytic matricoma	2	2.82%
Onycholemmal cyst	1	1.41%
Nail plate hemorrhage	1	1.41%
Melanocyte neoplasia		
Nail Matrix Lentigo	20	28.17%
Nail Matrix Nevus	12	16.90%
Nail Matrix Melanoma	1	1.41%
Infectious		
Onychomycosis	1	1.41%
Longitudinal erythronychia		
Age (Mean, SD)	55.36	15.08
Gender (*n*, %)		
Male	17	35.42%
Female	31	64.58%
Ethnicity (*n*, %)		
White	17	35.42%
Hispanic or Latino	29	60.42%
Black or African American	1	2.08%
More than one race	1	2.08%
Diagnosis (*n*, %)		
Onychopapilloma	20	41.67%
Verruca vulgaris	7	14.58%
Digital mucus cyst	3	6.25%
Fibrosis/scar	2	4.17%
SCC		
Invasive SCC	3	6.25%
SCCIS	2	4.17%
Glomus tumor	2	4.17%
Nail matrix scar	2	4.17%
Chronic dermatitis	3	6.25%
Pyogenic granuloma	1	2.08%
Subungual keratoma	1	2.08%
Onychomatricoma	1	2.08%
Normal nail with dystrophy	1	2.08%
Longitudinal pachyonychia		
Age (mean, SD)	53	23.22
Gender (*n*, %)		
Male	3	37.50%
Female	5	62.50%
Ethnicity (*n*, %)		
White	3	37.50%
Hispanic or Latino	5	62.50%
Diagnosis (*n*, %)		
Nonmelanocytic tumors		
SCC	1	11.11%
Onychomatricoma	6	66.67%
Onychocytic matricoma	2	22.22%

*SCC*, squamous cell carcinoma; *SCCIS*, Squamous cell carcinoma in situ.

**Fig 1:**
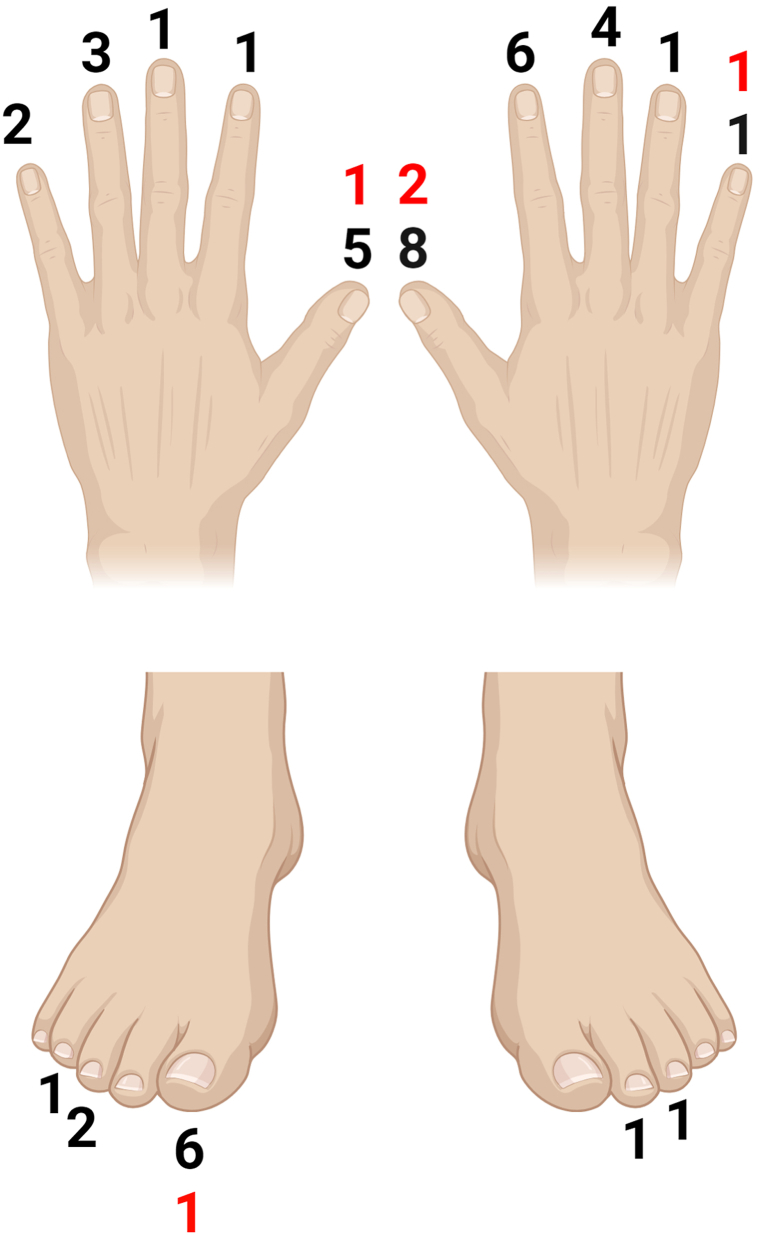
Schematic depiction of the digits involved in longitudinal erythronychia with benign etiologies in black and malignant etiologies in red.

This work represents one of the largest cohorts to date of biopsy-proven diagnoses for monodactylous nail plate change. Moreover, our cohort was multiracial, with over 50% of our patients identified as Hispanic or of mixed race. Previous studies have been limited by the absence of demographic data, homogenous cohorts, or low sample sizes. Although most cases were benign, we found higher rates of malignancy for LE than has previously been described.^2^ Detection of LE can be challenging, particularly in darker skin types, as the classic erythema associated with it is often absent or is replaced by leukonychia, nail splitting, or thinning of the nail plate. Of the 8 SCCs identified in our cohort, only 3 were in White patients, all of which presented as either LE or LPN. In non-White patients, 3 presented as LE and 2 as LM. This varying presentation of SCC as LM has previously only been described in case reports.^3^, ^4^, ^5^ Limitations of our study include its retrospective nature, the bias of only including biopsied cases, and being isolated to a single academic institution. We hope our data will inform physicians of the breadth of pathologies monodactylous nail changes represent and prepare them to perform or refer patients for nail matrix biopsy when indicated.

## Conflicts of interest

None disclosed.
